# Otovent Versus Valsalva: Physiological Insights for Diagnostic and Therapeutic Autoinflation in Eustachian Tube Dysfunction

**DOI:** 10.1002/ohn.70331

**Published:** 2026-07-03

**Authors:** Kiran Joshi, Victor Lim, Victor Ho, James Tysome, Matthew Smith

**Affiliations:** ^1^ School of Clinical Medicine University of Cambridge Cambridge UK; ^2^ Department of Otolaryngology Cambridge University Hospitals NHS Foundation Trust Cambridge UK

**Keywords:** autoinflation, diagnosis, Eustachian tube, Otovent, treatment, Valsalva

## Abstract

**Objectives:**

Autoinflation aids assessment of Eustachian tube function, or as a conservative treatment for obstructive Eustachian tube dysfunction. This study compares the physiological characteristics of 2 autoinflation techniques, the Otovent® device and the Valsalva manoeuvre, to inform clinical application for both diagnosis and therapy.

**Study Design:**

Prospective physiological study.

**Setting:**

Tertiary academic teaching hospital.

**Methods:**

Twenty‐one healthy adult volunteers performed 3 trials of both the Otovent and Valsalva manoeuvres. A linear mixed‐effects model (LMM) was used as the primary analysis to compare the mean maximum and plateau nasopharyngeal pressures. Intra‐subject repeatability was assessed using the intraclass correlation coefficient (ICC), while inter‐subject consistency was evaluated by comparing standard deviations. Eustachian tube (ET) opening rates were measured via tubo‐tympano‐aerodynamic‐graphy, otoscopy, and participant report.

**Results:**

The Valsalva manoeuvre generated significantly higher nasopharyngeal pressures. Mean maximum NP pressure for Valsalva (597.5 daPa) was greater than for Otovent (482.3 daPa) (*F*(1, 104) = 18.82, *P* < .001). This difference was more pronounced for plateau pressures (Valsalva: 486.0 daPa vs Otovent: 278.0 daPa; *F*(1, 104) = 126.42, *P* < .001). Conversely, the Otovent demonstrated markedly superior consistency, showing higher intra‐subject repeatability for plateau pressure (ICC = 0.872 vs 0.572) and substantially less intersubject variability (SD ± 39.8 daPa vs ±144 daPa). ET opening rates were comparable between the 2 techniques.

**Conclusions:**

Otovent facilitates autoinflation at lower but significantly more consistent pressures than Valsalva. Thus, Otovent may offer greater clinical value when a controlled, repeatable insufflation pressure is required for diagnostic or therapeutic purposes.

The Eustachian tube (ET) is a complex anatomical structure responsible for 3 critical functions: middle ear pressure regulation, mucociliary clearance, and protection of the middle ear from nasopharyngeal pathogens and sounds. Failure of these functions leads to Eustachian tube dysfunction (ETD), affecting approximately 1% of the adult population.^1^ The clinical sequelae of ETD are significant, ranging from symptoms of aural fullness, otalgia, and tinnitus to the development of chronic middle ear pathologies such as otitis media with effusion (OME), tympanic membrane retraction, and cholesteatoma.^2^

ETD can be divided into subtypes which may overlap in an individual, including patulous ETD (a persistently open tube), baro‐challenge‐induced ETD, and, most commonly, obstructive ETD (OETD), which is characterized by the failure of the tube to open adequately.^1^ The management of OETD typically begins with conservative, non‐invasive strategies, and progresses to minimally invasive interventions and surgery for refractory cases where symptoms are intrusive.^3^ A key development has been the evaluation and increasing adoption of Balloon Dilation of the Eustachian Tube (BDET), a minimally invasive surgical procedure that has been shown to produce clinically meaningful improvement in OETD symptoms.^4^

When considering OETD, the establishment of BDET as a therapeutic option has sharpened the focus on patient diagnosis and case selection for surgery, as well as the initial, conservative phase of management. Before a patient is selected for surgery, the diagnosis of OETD must be sufficiently certain.^5^ Autoinflation is widely used as a method of diagnosing OETD and as a treatment option to improve symptoms, although evidence of efficacy is mixed. This creates a clinical need to better understand this method. This study addresses this need by investigating the physiological characteristics of different autoinflation techniques.

Autoinflation, the self‐generation of positive nasopharyngeal pressure to passively open the ET, is a simple, non‐pharmacological intervention for OETD. Many patients attempt to use autoinflation to relieve aural fullness, and the technique is included in some clinical guidelines for managing conditions associated with OETD, most notably OME where there is some evidence of efficacy in children and adults.^6^ The 2 most frequently employed autoinflation techniques are the Valsalva manoeuvre and the use of a purpose‐built medical device, the Otovent®. Autoinflation also plays an important role in the diagnosis of OETD, allowing assessment of passive ET opening without specialist equipment.^7^ If a patient reports air entering the middle ear, “the ear popping,” or a clinician sees outward movement of the drum, the ET is confirmed as patent, at least at above‐physiological pressures.

Despite the widespread use of autoinflation, a significant clinical challenge persists: there is a lack of physiological data to guide clinicians on which technique is preferable for specific clinical goals. The application of the Valsalva manoeuvre, in particular, may be highly variable both between and within individuals. This variability introduces uncertainty into the clinical setting; a failure to open the ET during a Valsalva manoeuvre could represent true tubal obstruction or simply be a result of poor patient technique, thereby confounding both diagnostic assessment and therapeutic efficacy.

The primary rationale for this study is to address the existing gap in physiological evidence regarding common autoinflation methods. By characterizing and comparing the nasopharyngeal pressures generated by the Otovent device and the Valsalva manoeuvre, this study is designed to determine if one technique offers advantages in terms of maximum and plateau nasopharyngeal pressures or consistency, and whether any differences translate into variation in the rates of ET opening in healthy adults.

## Materials and Methods

### Subjects, Screening, and Baseline Assessments

Ethical approval was obtained from the University of Cambridge Human Biology Research Ethics Committee. Healthy adult volunteers were recruited from staff and medical student groups via advertisement. Potential participants underwent a screening process to exclude those with a history of otological disease, a recent upper respiratory tract infection (within 4 weeks), or known allergies to latex. Following written informed consent, further screening with otoscopic examination (NTE100 USB digital otoscope, Teslong Technology Limited) was performed to exclude individuals with middle ear effusion, tympanic membrane perforation, or significant retraction pockets. Tympanometry (226 Hz tone at 85 dB SPL, Titan IMP440; Interacoustics) was used to exclude participants with Jerger type B or C traces, indicative of middle ear fluid or significant negative pressure, respectively.^8^ All participants completed the 10‐item Cambridge Eustachian Tube Dysfunction Assessment (CETDA) to screen for and quantify symptoms of ETD.^9^ CETDA scores greater than or equal to 17 indicate a positive result for ETD.^9^ Selected patients did not fall outside the CETDA defined median (13) and IQR (3) for healthy individuals. Only one ear per subject was tested, based on participant preference. Figure 1 shows the study pathway.

**Figure 1:**
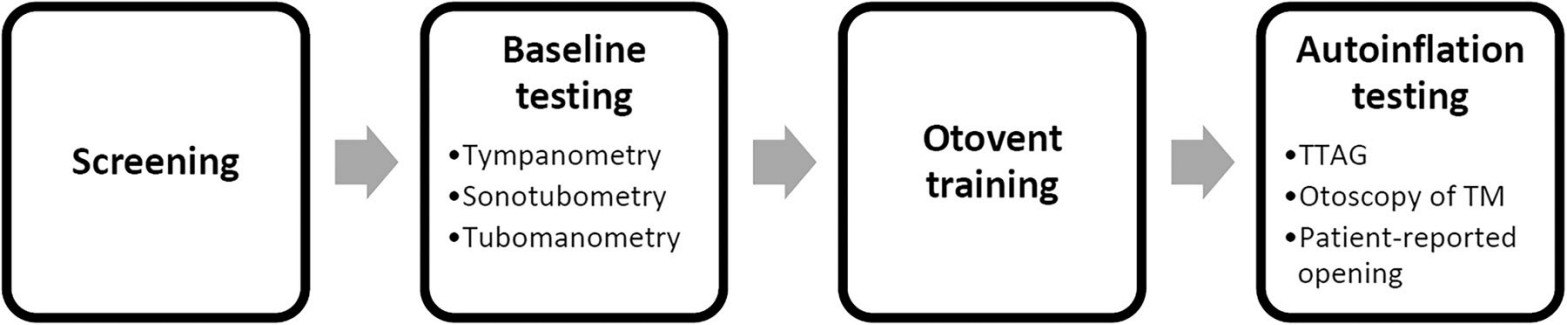
Testing schema, repeats were carried out back‐to‐back. TM, tympanic membrane; TTAG, tubo‐tympano‐aerodynamic‐graphy.

Baseline ET function was comprehensively assessed using a battery of standard tests previously characterized by the authors (Table 1).^7^ Tympanometry was performed 3 times to record mean middle ear pressure (MEP) and tympanic compliance. Sonotubometry (JK‐05A; Rion Co.) was used to measure sound transmission from the ipsilateral nostril to the external auditory canal (EAC) during 5 dry swallows; an increase of >5 dB was defined as ET opening. Finally, tubomanometry (TMM, Tubomanometer; Spiggle & Theis) measured pressure transmission from the nasopharynx to the middle ear during 3 wet swallows (swallowing water) against a constant nasopharyngeal pressure of +400 daPa; any pressure increase >0 daPa in the EAC was considered an objective ET opening.

**Table 1:** Methodology and Measured Outcome for Each Baseline Test of Eustachian Tube Function

Baseline test	Apparatus	Patient manoeuvre (repeats)	Measured outcome (units)
Tympanometry	Titan IMP440	At rest (3)	Middle ear pressure (daPa)
			Tympanic compliance (ml)
Sonotubometry	Rion JK‐05A	Swallow without water (5)	EAC sound (dB)
Tubomanometry	Spiggle & Theis Tubomanometer	Swallow with water (3)	EAC pressure change (daPa)

Abbreviation: EAC, external auditory canal.

### Experimental Protocol and Equipment

Otovent devices (Abigo Medical AB) consist of a plastic nosepiece that directs exhaled air into a calibrated latex balloon, providing visual feedback of successful pressure generation.^10^ The devices were modified by inserting a Luer lock adaptor into the nosepiece, allowing for connection to a digital pressure meter (Testo 510; Testo SE & Co. KGaA). For the Valsalva manoeuvre, NP pressure was recorded using a modified nasal probe from the JK‐05A sonotubometer, which was held within the pinched nostrils and connected in parallel to the same pressure meter.

Participants were instructed in the correct use of both techniques. For the Otovent, manufacturer guidance was followed, and each participant performed an initial unrecorded inflation to overcome the initial low compliance of the new balloon. For the Valsalva manoeuvre, participants were instructed to perform a forced exhalation against a closed mouth and pinched nostrils. Each participant performed 3 consecutive trials of each autoinflation technique. During each trial, they were asked to maintain the pressure at the end of the manoeuvre to allow for measurement of both a peak and a sustained plateau NP pressure.

ET opening during autoinflation was assessed using 3 distinct methods. First, Tubo‐tympano‐aerodynamic‐graphy (TTAG) was performed using the JK‐05A device to objectively measure pressure changes in the EAC, with a change >0 daPa considered indicative of ET opening. Second, video‐otoscopy was used to record the tympanic membrane during each manoeuvre, and any visible outward movement was subsequently judged by 2 testers independently scoring, as an objective sign of ET opening. Third, participants were asked to report any subjective sensation of aural pressure change or a “pop,” which was recorded as a subjective marker of ET opening.

### Statistical Analysis

All statistical analyses were conducted using IBM SPSS Statistics (Version 28.0).

The primary analysis of the continuous outcomes (maximum and plateau nasopharyngeal pressures) was performed using a linear mixed‐effects model (LMM). This approach was chosen to correctly account for the non‐independence of the 3 repeated trials nested within each participant. In the model, the autoinflation manoeuvre (Otovent vs Valsalva) was specified as a fixed effect, and a random intercept for each participant was included to model individual differences in baseline pressure generation. The normality of the model's residuals was assessed via visual inspection of Q‐Q plots. Model‐adjusted estimated marginal means were used to compare the average pressures between the 2 manoeuvres.

To specifically quantify trial‐to‐trial consistency, the intraclass correlation coefficient (ICC) was calculated for each manoeuvre. ICC estimates and their 95% confidence intervals were based on a two‐way mixed‐effects model assessing absolute agreement for average measures.

For dichotomous outcomes, the rates of ET opening observed during otoscopy, TTAG pressure change, and participant report were compared between the 2 techniques using McNemar's test, which is appropriate for paired categorical data. A *P* < .05 was considered statistically significant for all analyses.

## Results

### Participant Characteristics and Baseline Function

Twenty‐one healthy volunteers (8 female, 13 male; mean age 23 years) were recruited. None were excluded after consenting due to otoscopy or tympanometry screening. All participants successfully completed all phases of the study. The median CEDTA score for the cohort was 11 (interquartile range 10‐14), confirming the absence of significant ETD symptoms. Mean MEP and tympanic compliance were within the normal range for 100% and 95.2% of tested ears, respectively.^11^ Objective ET opening during baseline testing was demonstrated in 82.9% of participants with sonotubometry and 72.7% with tubomanometry; rates that are comparable to previously published data for healthy adults.^12^, ^13^, ^14^

### Nasopharyngeal Pressure: Magnitude and Consistency

The Valsalva manoeuvre generated significantly higher nasopharyngeal (NP) pressures than the Otovent device across both maximum and plateau phases (Figure 2). The LMM revealed a significant main effect of the manoeuvre on maximum NP pressure (*F*(1, 104) = 18.82, *P* < .001). The estimated mean maximum pressure for Valsalva was 597.5 daPa (95% CI [545.1, 649.9]), compared to 482.3 daPa (95% CI [429.9, 534.7]) for the Otovent device. This difference was even more pronounced for plateau pressures (*F*(1, 104) = 126.42, *P* < .001). The mean plateau pressure for Valsalva was 486.0 daPa (95% CI [445.9, 526.0]), significantly exceeding the 278.0 daPa (95% CI [237.9, 318.0]) produced by the Otovent device.

**Figure 2:**
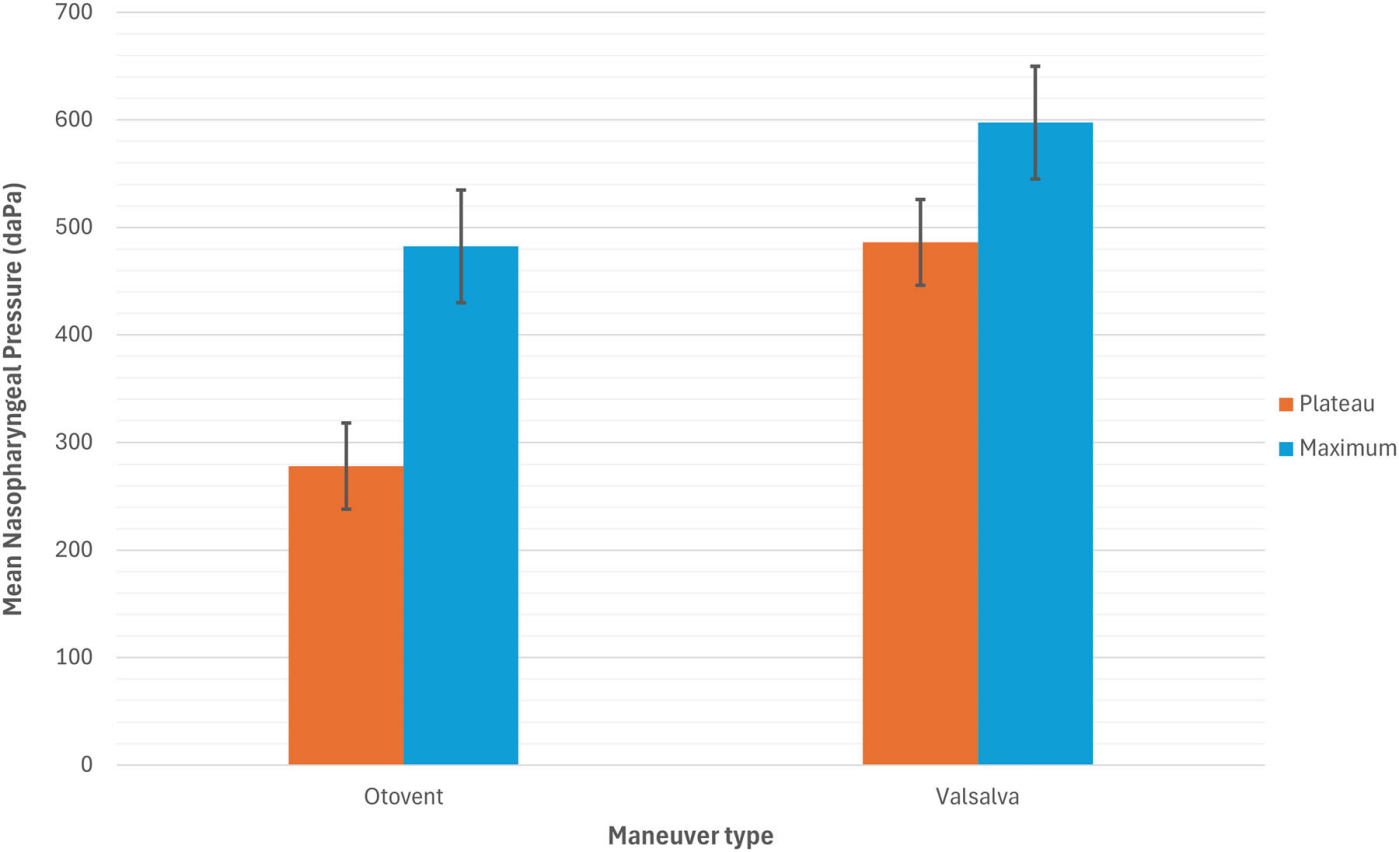
Comparison of mean nasopharyngeal pressures by autoinflation manoeuvre. The bars represent the estimated marginal means (±95% confidence interval shown as error bars) for both maximum and plateau pressures, derived from a linear mixed‐effects model.

While generating lower pressures, the Otovent device demonstrated markedly superior consistency, both within and between subjects. For intra‐subject repeatability, the ICC for plateau pressure was excellent for the Otovent (ICC = 0.872), whereas the Valsalva manoeuvre showed only moderate reliability (ICC = 0.572). These data are highlighted in Table 2.

**Table 2:** Intraclass Correlation Coefficient Estimates and 95% Confidence Intervals for Maximum and Plateau Nasopharyngeal Pressures in Valsalva and Otovent Manoeuvres

Nasopharyngeal pressure	Technique	Intraclass correlation coefficient (ICC)	95% Confidence interval	
			Lower bound	Upper bound
Maximum	Valsalva	0.633	0.403	0.813
	Otovent	0.694	0.480	0.849
Plateau	Valsalva	0.572	0.326	0.776
	Otovent	0.872	0.760	0.941

For inter‐subject consistency, the standard deviation of the plateau pressures was substantially smaller for the Otovent (SD ± 39.8 daPa) compared to the Valsalva manoeuvre (SD ± 144 daPa), indicating far less variability from person to person. The LMM analysis confirmed this finding, showing that the variance component attributable to the participant (a direct measure of inter‐subject variability) was significantly smaller for the Otovent condition.

### Eustachian Tube Opening Rates

Despite the significant differences in the magnitude and consistency of the generated nasopharyngeal pressures, the rates of successful ET opening were comparable between the 2 techniques. Table 3 shows no statistically significant differences in ET opening rates as measured by objective TTAG (*P* = 1.00), objective otoscopic visualization (*P* = .250), or subjective participant report (*P* = .625).

**Table 3:** Eustachian Tube Opening Rates With Valsalva and Otovent Use for Various Tests

Test	Measure	Technique	ET opening rate %	*P* value
TTAG	EAC pressure change	Valsalva	81.0	1.000
		Otovent	76.2	
Otoscopic examination	Movement detected	Valsalva	85.7	.250
		Otovent	71.4	
Subjective opening	Participant reported	Valsalva	76.2	.625
		Otovent	66.7	

Abbreviations: EAC, external auditory canal; TTAG, tubo‐tympano‐aerodynamic‐graphy.

## Discussion

### Principle Findings

This study provides a detailed physiological comparison in healthy adults of the 2 most common autoinflation techniques used in the assessment and management of OETD. Principal findings reveal a clear dichotomy: the Valsalva manoeuvre generates significantly higher, but more variable, nasopharyngeal pressures, whereas the Otovent device produces lower but substantially more consistent pressures, both within and between individuals. This was particularly evident for the sustained plateau pressure, where Otovent demonstrated good intra‐subject reliability and remarkably low inter‐subject variability. Despite these marked differences in pressure profiles, both techniques achieved statistically similar rates of ET opening in this cohort of healthy adults. These findings have implications for how these techniques should be selected and applied in the clinical setting, depending on the specific clinical goal.

### Diagnostic Utility for Autoinflation Techniques

For any diagnostic test, consistency and repeatability are important characteristics to produce a predictable outcome when applied under similar conditions. We found a wide range of pressures generated by different individuals performing a Valsalva manoeuvre, making consistent interpretation of ET opening difficult either within or between individuals. Suboptimal patient effort or technique could provide a false positive finding of OETD. This variability compromises its utility as a standardized diagnostic tool for OETD.

In contrast, the Otovent device demonstrated very high consistency, particularly in the plateau pressure it generates, which averaged ~280 daPa with a very small standard deviation. This could be expected as the pressure to maintain the balloon a certain size is inherent to the balloon material, and the balloon provides a visual “pressure gauge” for the participant inflating it. This suggests that autoinflation augmented by the Otovent could function as a standardized, clinical test for passive ET opening, and OETD in general. A further advantage is that the balloon provides the clinician with an indication of the pressure that is being generated. A clinician could instruct a patient to inflate the Otovent balloon while observing the tympanic membrane for movement, confident that a known and relatively constant pressure is being delivered to the nasopharynx. This transforms the assessment from a qualitative “can the patient pop their ears?” to a semi‐quantitative challenge.

Work is needed to explore the 2 tests in populations with OETD, and the merits of their differing pressures. For tubomanometry, nasopharyngeal pressures of 300 and 400 daPa are considered of most diagnostic value, distinguishing affected form healthy ears, although this test evaluates both active (muscle‐assisted) and passive functions of the ET.^7^ In contrast, the forced response test specifically measures passive ET opening pressure, with the literature reporting opening pressures in healthy adult ears in the range 300 to 350 daPa (≈30‐35 cm H_2_O).^15^, ^16^ In our previous assessment of test diagnostic ability for OETD, the Valsalva manoeuvre consistently had a sensitivity and specificity both approximating 75%,^7^ in theory suggesting a balance of false positive and false negative results. In our current study, the mean nasopharyngeal pressure using the Otovent device was 482 ± 52 daPa at peak and 278 ± 40 daPa for the plateau. The lower plateau pressures generated by Otovent may provide an adequate stimulus for passive ET opening in most healthy ears, as supported by the identified opening rate. However, it is also possible that Otovent use may lead to false negative results if adopted in diagnosis, perhaps supported by the observed trend toward higher ET opening rates when using the Valsalva manoeuvre.

With the increasing use of interventions like BDET, the ability to objectively identify and confirm obstructive dysfunction is important for appropriate patient selection. With further characterisation in OETD‐affected ears, the Otovent device could serve as a simple, inexpensive, and readily available tool to improve the accuracy of the clinical diagnostic pathway for OETD, in the place of Valsalva‐based tests. Repeatability is also of value in outcome measurement, permitting reliable measurement of pretherapy and posttherapy ET opening under the same conditions.

Although few studies have directly measured nasopharyngeal pressures during autoinflation, the existing literature provides some context for our findings. For the Otovent device, Marshall et al measured the mechanical inflation pressure of the balloon at approximately 93 mmHg (≈124 daPa) using a pressure monitor, though this reflects the intrinsic resistance of the balloon material rather than the in vivo nasopharyngeal pressures reported in our study.^10^ For the Valsalva manoeuvre, Swarts et al reported a mean nasopharyngeal pressure of 707 ± 318 daPa (range 200‐1153 daPa) in healthy adults undergoing ET function testing, broadly consistent with our finding of 598 daPa and notably demonstrating similarly wide inter‐subject variability.^17^ Our measured nasopharyngeal pressures for both Otovent (482 daPa peak, 278 daPa plateau) and Valsalva (598 daPa peak, 486 daPa plateau) substantially exceed the 30 to 50 mmHg (≈40‐67 daPa) widely cited as the minimum required for an effective Valsalva manoeuvre,^10^ suggesting that both techniques generate pressures well above the threshold for passive ET opening in healthy ears. To our knowledge, no prior study has directly compared the in vivo nasopharyngeal pressures produced by the Otovent device and the Valsalva manoeuvre, underscoring the novelty of the present findings.

### Therapeutic Utility

Autoinflation has some evidence of a beneficial effect on OME‐specific quality of life and persistence of OME in the short term in children, though the aetiology of OME in children is complex and not just related to ET opening.^18^, ^19^ While some patients with OETD report symptom relief with autoinflation, there is no evidence that repeated autoinflation directly affects the ability of the ET to open.

The therapeutic application of autoinflation is aimed at passively opening an obstructed ET to equalize middle ear pressure. Higher pressures generated by the Valsalva manoeuvre would likely be superior for therapeutic use, particularly in diseased states where the ET passive opening pressure is likely to be pathologically elevated. Reported opening pressures in ears with OETD can range from 320 to 740 daPa, a range that is more closely approximated by the pressures achieved with Valsalva.^16^, ^20^, ^21^ However, the fact that Valsalva makes an effective test for OETD dictates that many OETD affected ears will still not open with Valsalva.^7^

### Clinical and Research Implications

The findings from this study offer relevance for both clinical practice and future research. For clinicians, the results suggest a goal‐oriented approach to selecting an autoinflation technique. When the objective is diagnostic, to assess whether the ET can open to a passive pressure stimulus, the Otovent device should be considered as a standardized test due to its high consistency. When recommending therapeutic autoinflation, clinicians should counsel patients that although the Valsalva manoeuvre generates higher pressures, a device‐based method like the Otovent offers a more controlled and repeatable stimulus. This is of particular value in children, where Valsalva can be difficult to instruct and perform with adequate pressure generation. Some adults also find high‐pressure Valsalva to be uncomfortable and refrain from its use.

For researchers, this study highlights the need for further investigation into the long‐term therapeutic effects of different autoinflation profiles, and the effect of these on ET opening pressures over time. A prospective, randomized trial directly comparing a high‐pressure, variable technique (Valsalva) against a moderate‐pressure, consistent technique (Otovent) in a cohort of patients with confirmed OETD is warranted. Key outcomes for such a study should include not only short‐term symptomatic relief, as measured by validated patient‐reported outcome measures like the CETDA or ETDQ‐7, but also long‐term functional outcomes, such as changes in tympanometry and, critically, the need for subsequent procedural intervention like BDET.^5^

### Strengths and Limitations

This study has several strengths, including a robust within‐subjects design that allowed for direct comparison of the 2 techniques in each participant, minimizing inter‐individual confounding. The use of multiple, independent measures of ET opening—including objective instrumental (TTAG), objective observational (otoscopy), and subjective patient‐reported methods – provides a comprehensive assessment of functional outcomes.

The primary limitations of this study must also be acknowledged. The sample size of 21 participants is relatively small, and the findings should be validated in larger cohorts. The study was also conducted exclusively in a population of healthy young adults and therefore, the pressure profiles observed may not be directly generalizable to a patient population with OETD, or to other patient populations such as children.^18^

## Conclusions

Valsalva and Otovent produce distinct physiological pressures during autoinflation. The choice between them should be guided by the specific clinical goal. The Valsalva manoeuvre generates higher but more variable nasopharyngeal pressures, potentially suiting it to therapeutic applications, where it can be adequately performed. In contrast, the Otovent device produces a lower but remarkably consistent pressure stimulus, positioning the technique as a potentially valuable tool for the standardized diagnostic assessment of passive ET function, and outcome monitoring, an area of growing importance.

## Author Contributions

**Kiran Joshi**, study design, data collection, data analysis and manuscript preparation; **Victor Lim**, data analysis and manuscript preparation; **Victor Ho**, data collection and data analysis; **James Tysome**, study conceptualization, supervision, and manuscript review; **Matthew Smith**, study conceptualization, supervision, and manuscript review.

## Disclosures

### Competing interests

J.T. and M.S. received funding and a tubomanometer loan from Spiggle & Theis. The Cambridge Hearing Trust funded the Rion JK‐05A. Supported by the Cambridge Biomedical Research Centre. No other conflicts.

### Funding source

None.

## References

[1] Hamrang‐Yousefi S , Ng J , Andaloro C. Eustachian tube dysfunction. StatPearls. Published online February 13, 2023. Accessed August 25, 2025. 32310368

[2] Smith ME , Tysome JR . Tests of Eustachian tube function: a review. Clin Otolaryngol. 2015;40(4):300‐311. 10.1111/coa.12428 25851074

[3] Llewellyn A , Norman G , Harden M , et al. Interventions for adult Eustachian tube dysfunction: a systematic review. Health Technol Assess (Winchester, England). 2014;18(46):1‐180. 10.3310/HTA18460 PMC4781384 25029951

[4] Swords C , Smith ME , Patel A , Norman G , Llewellyn A , Tysome JR . Balloon dilatation of the Eustachian tube for obstructive Eustachian tube dysfunction in adults. Cochrane Database Syst Rev. 2025;2025(2):CD013429. 10.1002/14651858.CD013429.pub2 PMC11863300 40008607

[5] Smith ME , Bance ML , Tysome JR . Advances in Eustachian tube function testing. World J Otorhinolaryngol Head Neck Surg. 2019;5(3):131‐136. 10.1016/j.wjorl.2019.08.002 PMC6849358 31750424

[6] Perera R , Glasziou PP , Heneghan CJ , McLellan J , Williamson I . Autoinflation for hearing loss associated with otitis media with effusion. Cochrane Database Syst Rev. 2013;5:CD006285. 10.1002/14651858.CD006285.pub2 PMC11357689 23728660

[7] Smith ME , Takwoingi Y , Deeks J , et al. Eustachian tube dysfunction: a diagnostic accuracy study and proposed diagnostic pathway. PLoS One. 2018;13(11):e0206946. 10.1371/JOURNAL.PONE.0206946 PMC6224095 30408100

[8] Jerger J . Clinical experience with impedance audiometry. Arch Otolaryngol Head Neck Surg. 1970;92(4):311‐324. 10.1001/ARCHOTOL.1970.04310040005002 5455571

[9] Smith ME , Cochrane IL , Donnelly N , Axon PR , Tysome JR . The performance of patient‐reported outcome measures as diagnostic tools for eustachian tube dysfunction. Otol Neurotol. 2018;39(9):1129‐1138. 10.1097/MAO.0000000000001931 30106847

[10] Marshall K , Perera R , Glasziou P , Fleming S . Can party balloons replace autoinflation balloons to treat glue ear? A technical comparison. BJGP Open. 2021;5(3):BJGPO.2020.0178. 10.3399/BJGPO.2020.0178. PMC8278505 33589466

[11] British Society of Audiology . Technical Note: Recommended procedure for tympanometry. Br J Audiol. 1992;26(4):255‐257. 10.3109/03005369209076644 1446189

[12] Di Martino EFN , Nath V , Telle A , Antweiler C , Walther LE , Vary P . Evaluation of Eustachian tube function with perfect sequences: Technical realization and first clinical results. Eur Arch Oto‐Rhino‐Laryngol. 2010;267(3):367‐374. 10.1007/S00405-009-1074-9 19727790

[13] Smith ME , Blythe AJC , Baker C , Zou CC , Hutchinson PJA , Tysome JR . Tests of eustachian tube function: the effect of testing technique on tube opening in healthy ears. Otol Neurotol. 2017;38(5):714‐720. 10.1097/MAO.0000000000001375 28306652

[14] Schröder S , Lehmann M , Korbmacher D , Sauzet O , Sudhoff H , Ebmeyer J . Evaluation of tubomanometry as a routine diagnostic tool for chronic obstructive Eustachian tube dysfunction. Clin Otolaryngol. 2015;40(6):691‐697. 10.1111/COA.12451 25925071

[15] Moon JB , Swanson SA . Passive eustachian tube opening pressure: its measurement, normal values, and clinical implications. Arch Otolaryngol Head Neck Surg. 1983;109(6):364‐368. 10.1001/ARCHOTOL.1983.00800200010004 6847494

[16] Doyle WJ , Swarts JD , Banks J , Casselbrant ML , Mandel EM , Alper CM . Sensitivity and specificity of eustachian tube function tests in adults. JAMA Otolaryngol Head Neck Surg. 2013;139(7):719. 10.1001/JAMAOTO.2013.3559 PMC3792585 23868429

[17] Swarts JD , Alper CM , Mandel EM , Villardo R , Doyle WJ . Eustachian tube function in adults without middle ear disease. Ann Otol Rhinol Laryngol. 2011;120(4):220‐225. 10.1177/000348941112000401 PMC3616372 21585150

[18] Schilder AGM , Chonmaitree T , Cripps AW , et al. Otitis media. Nat Rev Dis Primers. 2016;2(1):16063. 10.1038/NRDP.2016.63 PMC7097351 27604644

[19] Webster KE , Mulvaney CA , Galbraith K , et al. Autoinflation for otitis media with effusion (OME) in children. Cochrane Database Syst Rev. 2023;9(9):015253. 10.1002/14651858.CD015253.PUB2 PMC10521168 37750500

[20] Alper CM , Teixeira MS , Swarts JD . Eustachian tube function in adults with ventilation tubes inserted for otitis media with effusion. J Int Adv Otol. 2018;14(2):255‐262. 10.5152/IAO.2018.4521 PMC6354449 30256199

[21] Kapadia M , Tarabichi M . Feasibility and safety of transtympanic balloon dilatation of Eustachian tube. Otol Neurotol. 2018;39(9):e825‐e830. 10.1097/MAO.0000000000001950 30124616

